# The Spatial Interaction Effect of Green Spaces on Urban Economic Growth: Empirical Evidence from China

**DOI:** 10.3390/ijerph191610360

**Published:** 2022-08-19

**Authors:** Hailing Zhou, Yan Liu, Miao He

**Affiliations:** 1 School of Political Science and Public Administration, Shandong University, Qingdao 266200, China; 2 School of History, Beijing Normal University, Beijing 100875, China; 3 School of Marxism, Shandong University, Jinan 250100, China

**Keywords:** spatial interaction, economic growth, spillover effect, green space rate of the built-up area

## Abstract

This paper measures the impact of urban green space construction rate on urban economic growth from the perspective of spatial interaction. To this end, we collect panel data of 31 provincial capital cities in China from 2001 to 2020 and use spatial economics models for empirical testing. The research results are summarized as follows: the level of green space construction can attract talents and investment by improving the environmental level of the city, and these financial expenditures, foreign investment, and talents are conducive to urbanization, thus having a significant positive impact on urban economic development. In addition, it also has a significant positive spatial spillover effect. In addition, the construction of urban green space will also stimulate the environmental protection of neighboring cities, which has a significant positive spatial dependence. At this time, talents and investment are affected by the environmental construction of neighboring cities, and the economic development of the city has also been significantly improved. The spatial spillover effect of green space construction on the economic level of surrounding cities is also positive. The empirical conclusions provide references for implementing green development strategies and promoting high-quality economic development.

## 1. Introduction

The continuous development of the human scientific and technological civilization and the expansion of urbanization have aggravated the often detrimental impact on the natural ecological environment. The resulting challenges for the human living environment are serious. As the main gathering places of humans, cities face particularly prominent problems, such as overheating, because of the heat island effect and pollution in the form of atmospheric smog. To address these problems, China has put forward an ecological garden city construction system with Chinese characteristics. In this system, urban green space construction—one of the important components of the creation of ecological garden cities—plays an indispensable role in the construction and development of ecological garden cities in China [1].

A city is a huge and complex system integrating ecology, economy, and society. A close relationship of interdependence and mutual restriction exists among its various subsystems. Ecology and the environment are important foundations and spatial carriers of the sustainable development of human society, economy, and culture. With the continuous transformation of economic growth patterns, urban economic development increasingly depends on systems such as information, management, and ecology. Together, these are referred to as the “soft” factor. According to research, the relative importance ratio of the four factors affecting urban ecology (i.e., geography, climate, pollution, and green space) is 34:66:25:25 [2]. The ecological economic function of the green space system should receive more research attention.

As an important category of urban ecosystems, urban green space refers to urban land with natural vegetation and artificial vegetation as the main forms [3]. It has become increasingly important to measure the state of harmony within the human–land relationship and the quality of life of residents [4]. Research on the interaction mechanism between urban green space and the economic development level is one aspect of the research on the relationship between the ecological environment and the accumulation of social wealth. Research in this field will deepen the understanding of the social–economic–natural complex ecosystem, thus enriching the practical basis for the construction of the ecological civilization.

At present, research on the economic effect of urban green spaces mainly focuses on evaluating the use value of urban green spaces [5]. The conditional valuation method and hedonic price method are commonly used by studies focusing on the monetization of the urban green space ecosystem service value [6]. To calculate the economic value of urban green spaces, scholars have compared the evapotranspiration cooling function of these spaces with the cooling function (and associated costs) of air conditioners [7]. To evaluate the economic effect of such urban green spaces, their value needs to be evaluated in monetary terms, which can then be used to estimate the public welfare created by urban green spaces as a public good. However, such an estimation does not directly reflect the interactive relationship between urban green spaces and economic development. In contrast, the real estate value-added effect of urban green spaces reflects the relationship between urban green spaces and economic development directly. The hedonic value model [8], semi-logarithmic hedonic price model [9], and geographically weighted regression have been used to explore the relationship between urban green spaces and real estate prices. The results showed that urban green spaces significantly increase the real estate value of residential areas nearby. The real estate value-added effect of urban green spaces confirms the existence of a correlation between urban green spaces and economic development [10]. However, the real estate market is only one of several components of the urban economy, and the relationship between urban economic development and urban green spaces needs to be discussed in detail at the macro level. Liao and Li [11] constructed a theoretical framework of urban green coordinated development in the field of labor in urban green technology innovation. They then used this framework to empirically test the impact of urban green technology innovation process specialization on urban green development. Artmann et al. [12] explored the current state of the art in developing, testing, and implementing multi-dimensional ecological, economic, social, and multi-scale regional, city, and neighborhood metrics that characterize urban sprawl and compact green cities. Using cross-sectional data of 283 Chinese cities in 2014, Li et al. [13] analyzed the promotion effect economic development has on urban greening using a spatial regression model. The results showed that per capita gross domestic product (GDP) has a positive impact on the urban green space rate.

In general, most studies at the macro level used regression analysis, and most of the results merely uncover the correlation between urban green spaces and economic development. Whether a causal relationship exists between urban green spaces and economic development, and whether this causal relationship is based on economic development affecting urban green spaces or urban green spaces affecting economic development (or a two-way causal relationship) needs further exploration. In this study, the spatial econometrics model is comprehensively used to test the causal relationship between urban green spaces and economic development. Furthermore, the spatial impact logic of both is also explored.

## 2. Analysis of the Economy Promoting Function of Green Spaces

Urban green spaces are an important part of the urban ecosystem, which includes various types and scales of garden green spaces that yield social, economic, and environmental benefits in cities [14]. Promoting the coordinated development of urban nature, economy, and society is of positive significance. As a subsystem of the urban composite system, green space construction and other subsystems interact and form a virtuous circle. Before the 1970s, many countries and regions adopted countermeasures, mostly including the use of legal, administrative, engineering, and technical measures to control pollution [2]. Since the 1980s, urban planning is gradually developing toward ecology, focusing on urban ecological planning and rational layout. Especially over the past 15 years, urban construction based on ecological requirements has flourished in European and North American cities, and all such efforts emphasize the impact of green space systems on living space [15]. The ecological economic value of various economic activities in a city and the construction of ecological green spaces have become the standard for measuring the degree of civilization of a city and its level of urban development.

**Figure 1:**
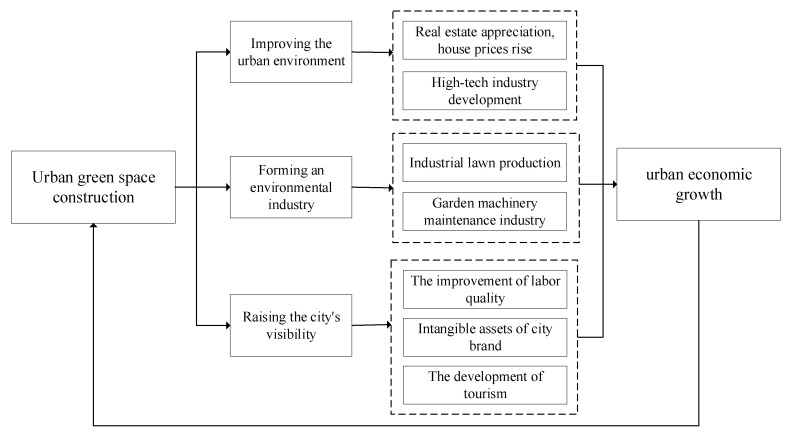
Analysis of the promotion function of green space to economy.

Relevant studies have demonstrated that green space construction and urban economic development interact and form a virtuous circle [16]. The economic development level of a city is an important factor affecting the construction of its green space system, providing a good social foundation and economic guarantee for the construction of the green space. The construction of the green space system is related to the spatial layout and functional zoning of the entire city, which, in turn, also affect the economic pattern of the city. A high-quality green space system can promote the coordinated development of both the environment and the economy, improve the function of the city’s composite system, and thus enhance the economic development level of the entire city. The mechanism with which urban green space construction promotes economic growth is depicted in Figure 1.

### 2.1. Transforming Environmental Advantages into Economic Advantages

High-quality green space construction can greatly improve the environmental quality of the city, create regional environmental advantages, and promote the appreciation of land prices between urban areas and various economic components in the city layout. Urban green construction can promote the appreciation of urban land prices and various economic components in the layout [17]. The urban environment is both the material basis for economic development and its space carrier. Most cities face the “stubborn diseases” left by the traditional extensive economic growth mode and the industry-oriented urban layout of modern cities. Starting from the treatment of three wastes and industrial transformation, high-standard environmental protection pressure and strong industrial inertia must be overcome. As the various industries within the industrial system are interconnected, it is ineffective to transform only one or a few enterprises alone. Transforming the entire industrial system is equivalent to readjusting the economic structure and reconstructing urban layout. Such a project is huge, and the investment of original resources is wasted. In contrast, if urban green space construction is used as a breakthrough point to carry out urban ecological construction, the required investment, as well as scientific and technological strengths, are relatively small. The improvement of the city image happens immediately, and the visual effect is clear (from point to line and from line to surface). Fueled by the scale effect, a city can also learn from the advanced experience of ecological regulation of developed countries. Thus, the urban spatial layout can be optimized, regional comparative advantages in the urban image strategy can be established, and a beneficial natural foundation and space carrier for urban economic development can be created [18].

Urban greening can also improve the urban investment environment and attract foreign capital investment. The investment environment of a city includes many factors, such as policy, location, and economy; the urban environment is one of the most basic conditions [19]. Advances in the transportation network and information transmission technology have greatly accelerated the temporal and spatial allocation of material resources and knowledge information. Thus, the location differences between major cities have gradually been eliminated. The policy advantage of being an early coastal city has also gradually weakened. In contrast, a high-quality urban environment can attract foreign capital investment, promote the continuous renewal of economic quality and structure, and become an important source of urban economic growth.

The development and agglomeration of high-tech industries will drive the optimization and upgrading of the entire urban industrial structure. Since entering the era of the knowledge economy, the high-tech industry has become the fastest growing and most vital industry [20]. The rapid development of this industry can realize the transformation of the urban economic growth mode and drive the optimization and upgrading of the industrial structure [21]. Measuring the foundation and stamina of a city’s economic development largely depends on the degree of development of its high-tech industry. In contrast to the heavy dependence of traditional economic industries on natural resources and transportation conditions, the development of high-tech industries imposes higher requirements on external environmental conditions. This leads to the tendency of knowledge-intensive industries (such as high-tech and information services) to concentrate in cities with apparent environmental advantages.

### 2.2. Urban Green Space Construction Creates a New Economic Industrial Chain

The urban green space system, urban transportation facilities, as well as post and telecommunication facilities, are all part of the urban infrastructure [22]. According to the World Bank Development Report, the construction of urban public facilities and the development of economic industries are growing simultaneously. Every percentage point increase in the stock of public facilities will also increase the GDP by one percentage point. The average yield of the urban infrastructure reaches 16%. As urban public construction moves toward industrialized operation, green space construction—an indispensable and important industry for urban economic development—is regarded as an organic part of the urban economic industry. Because the size and growth of the output share of urban greening and the urban construction industry are not only directly related to the total economic volume of the city, they also affect the industrial layout of the city, as well as the development of other industries through the industrial chain. Therefore, green space construction, as a high-efficiency economic activity, has also been incorporated into the virtuous circle of urban economic development. Through the systematic development of various industries covered by urban green space construction and the industrial chain affected by green space construction (such as lawn industrial production, construction and development of gardens, and machinery maintenance production), new industrial groups can be created and a complete industrial system can be established [23]. At the average world development level, the rate of return of urban public services, including urban green space construction, can reach 25%. Urban green space construction is indeed an attractive growth path for the urban economy and plays an irreplaceable role in stimulating urban economic demand and urban economic growth.

### 2.3. Improving City Popularity and Providing Intangible Capital for Economic Development

City popularity is a concentrated external reflection of a city’s comprehensive strength and an intangible capital support for the economic development of the city [24]. First, green space construction and the continuous improvement of the urban environment increase a city’s popularity, form the intangible asset of the city’s brand, and promote the steady development of the city’s tourism industry [25]. Second, urban green space construction also attracts high-quality talents both from China and internationally and optimizes the allocation of urban human resources [26]. Attracting capital and talents will likely lead to the generation of projects and the start of businesses. Further positive impacts are the increasing intensity of the city’s introduction of intellectual capital, the formation of a gathering place for intellectual introduction and international capital, and the associated gathering of many high-level talents. Moreover, attracting capital transforms the advantages of human resources into economic advantages, improves the competitiveness of the city, and ultimately greatly promotes the development of the city’s economy [27].

## 3. Materials and Methods

In this section, an overview of the selection and treatment of research variables, the selection and design of the empirical model, and the data sources is presented. In this paper, a spatial econometric model is employed to empirically test the spatial relationship between the growth of the urban economy and the construction of urban green spaces.

### 3.1. Variables

(1) Explained variable: Urban economic growth is measured by regional GDP [28]. To truly reflect economic growth, the real GDP is used after eliminating price factors based on 2001.
(2) Core explanatory variables: The green space rate of the built-up area is selected as the core research index for the following three reasons [29]: first, from the connotation of the index, this index is one of the core indicators for describing the level of urban green space construction. It is a relative index with strong comparability. Second, at the management level, this indicator is important for governmental departments to prepare urban construction planning and assess “ecological garden cities” and “garden cities”. Third, at the technical level, this indicator is an official statistical indicator, which has the unity and continuity of inter-city panel data.
(3) Control variables: For the built-up area [30], indicators for measuring urban land use mainly include the area of built-up area, governmental expenditure, and foreign direct investment. Among them, the built-up area is measured by the municipal public utility area and the basic public facility area, the government expenditure is measured by the payment by the government to perform its functions and obtain the required goods and services, and the foreign direct investment is measured by the funds borrowed by the state-owned enterprises from abroad. For the labor force quantity [31], the total employment was selected to measure the urban labor factor input, and the level of human capital is characterized by the average years of education [32]. The urbanization level is measured by comparing the urban population with the total population at the end of the year [33].

The relevant variables studied in this paper are explained in Table 1.

### 3.2. Data Sources

Limited by data availability, the research sample comprised 31 provincial-level administrative cities in China. The study time interval was selected from 2001 to 2020. The data were obtained from the 2002–2021 China Statistical Yearbook, the China City Statistical Yearbook, the China Urban Construction Statistical Yearbook, and the 2002–2021 Statistical Bulletin on National Economic and Social Development. Missing data were interpolated using linear interpolation. In addition, considering the data dimension and to reduce heteroscedasticity, the modeling data were logarithmized.

### 3.3. Subsection

#### 3.3.1. Spatial Autocorrelation Test

To explore whether spatial autocorrelation exists between lnGDP and UGR, the global Moran’s I index is tested at the urban economic growth and green space rate of 34 provincial-level administrative cities in China from 2001 to 2020. The global Moran’s I is calculated as follows:

(1) $$Moran\prime s\;I=\frac{\sum_{i}^{n}\sum_{j}^{n}W_{ij}(y_{i}-\bar{y})(y_{j}-\bar{y})}{S^{2}\sum_{i}^{n}\sum_{j}^{n}W_{ij}}$$

where $S^{2}=\frac{1}{n}\sum_{i}^{n}{\left(y_{1}-\bar{y}\right)}^{2}$, $\bar{y}=\frac{1}{n}\sum_{i}^{n}y_{i}$. n represents different cities, and $W_{ij}$ represents the spatial weight matrix. The row-standardized classical 0–1 matrix is used, as shown in Equation (2).

(2) $$W_{ij}=\left\{ \begin{array}{l} 1\;the\;region\;i\;is\;adjacent\;to\;the\;region\;j\\ 0\;the\;region\;i\;is\;not\;adjacent\;to\;the\;region\;j \end{array}\right.\;$$

The value of the Moran’s I index is between [−1, 1]. A Moran’s I greater than zero means that similar observations show positive spatial autocorrelation in the study area, and there is a spatial aggregation effect among variables. A Moran’s I of less than zero means that similar observations show positive spatial autocorrelation in the study area, and there is a spatial aggregation effect among variables.

#### 3.3.2. Measuring Spatial Spillover Effect for Model Construction

To measure the impact urban green space construction has on the economic growth of local and surrounding areas, referring to the spatial model constructed by Xu, X.X. and Wang, Y.H. (2017) [34], the spatial Durbin model (SDM) is used in this paper. In addition, both a spatial lag model (SAR) and a spatial error model (SEM) are constructed to illustrate the choice of spatial econometric models and related tests. To overcome possible endogenous problems of the model, this paper uses the time lags of all explanatory variables as proxy variables. From this, the spatial econometric model is obtained as shown in Equations (3)–(5).

SAR, shown in Equation (3), is mainly used to study the spatial spillover effect of urban economic growth on the surrounding areas.

(3) $$lnGDP_{it}=\beta WGDP_{i}+\beta_{1}WUGR+\beta_{2}UGR+\beta_{i}control+\mu_{i}+v_{t}+\varepsilon_{it}$$

SEM, shown in Equation (4), is mainly used to study the spatial interaction effect of the missing items in the modeling process.

(4) $$\begin{array}{l} lnGDP_{it}=\beta_{1}WUGR+\beta_{2}UGR+\beta_{i}control+\mu_{i}+v_{t}+\varepsilon_{it}\\ \varepsilon_{it}=\lambda W\varepsilon_{it}+\varphi_{it}\;\varphi_{it}\sim N(0,\sigma_{it}^{2}) \end{array}$$

SDM, shown in Equation (5), considers both the spatial lag items of explanatory and explanatory variables.

(5) $$\begin{array}{cc} lnGDP_{it}= & \beta WlnGDP_{i}+\beta_{1}WUGR+\beta_{2}UGR+\beta_{i}control+\\ & \theta_{i}Wcontrol_{it}+\mu_{i}+v_{t}+\varepsilon_{it} \end{array}$$

## 4. Results and Discussion

### 4.1. Time and Space Changes of Economic Growth and Green Space Construction

We analyzed the spatial evolution characteristics of urban green space construction and economic growth in 2001 and 2020, as shown in Table 2. To further realize the degree of change in each city, we categorize it according to the degree of growth as low–medium–high.

Table 3 and Table 4 show the spatial evolution characteristics of urban green space construction and economic growth in the years of 2001 and 2020. Table 2 shows that the spatial pattern changes from “East > Center > West” to “East > West > Center”. This shows that the hot spots of the green space rate in the built-up area are concentrated in the east, while the cold spots have migrated from western and central regions to the east, showing clear spatial evolution characteristics. Specifically, Nanjing and Beijing have always been the vanguards of urban green space construction. Guangzhou, Hohhot, Jinan, Guiyang, and other eastern cities have continuously improved their level of urban green space construction.

Table 2 shows that the economy of eastern cities has always been more developed, and cities such as Beijing, Shanghai, Nanjing, and Hangzhou have always been important economic centers. However, the urban economy in the northeastern region is relatively weak, and cities such as Lhasa, Hohhot, Yinchuan, and other urban economies are relatively weak. Cities in the central and western regions have relatively large development potential. Example cities are Hefei, Chongqing, Guiyang, and Nanning. Therefore, central and western cities will likely be the backbone of future economic development.

### 4.2. Spatial Autocorrelation Test

Using Equations (1) and (2), the global Moran’s I for the past decade was calculated (Table 4). Since 2001, the global Moran’s I data of the explained variable lnGDP and the explained variable UGR have passed the significance test, and both showed positive autocorrelation. This implies that the green space rate and urban economic growth in urban built-up areas are not randomly distributed but are significantly spatially agglomerated.

From the perspective of the time evolution of economic growth, from 2001 to 2004, the Moran’s I value of the explained variable increased from 0.275 to 0.298, and the spatial correlation of urban economic growth also increased. From 2005 to 2010, the Moran’s I value remained relatively stable and followed a downward trend but at a slow rate. During this period, China’s urban economic growth level was generally low, and the development was relatively slow. From 2011 to 2020, the Moran’s I value increased steadily, and the spatial correlation also increased. This indicates that the economic growth of each city was relatively stable during this period, and the economy of economically developed cities spilled over to the surrounding areas. Consequently, the gap between the economic levels of cities gradually narrowed.

From the perspective of the time evolution of the urban green space construction level, from 2001 to 2010, the Moran’s I value of the explanatory variable fluctuated greatly. In this period, the spatial correlation was unstable, indicating that cities increasingly focused on urban green space construction. The promulgation and implementation of relevant policies and regulations, as well as the increased investment in landscaping fixed assets by governments at all levels, have promoted the rapid development of the green space rate in built-up areas. However, the rate of increase in the green space rate of built-up areas in key cities far exceeds that of surrounding cities, creating a gap between cities. This is known as the phenomenon of widening differences. From 2011 to 2020, the Moran’s I value was relatively stable and followed an upward trend but at a slower rate. During this period, the level of green space in urban built-up areas was generally high and in a state of low level and balance.

### 4.3. Analysis of Spatial Spillover Effects

To test the applicability of the model, spatial regression analysis was conducted on the three models of SDM, SAR, and SEM. The results are shown in Table 5. The regression results indicate that both the Wald and LR tests reject the null hypothesis. The SDM-adjusted R^2^ is 0.688 and the log-likelihood value is 760.554, which are significantly larger than the corresponding values of the SAR and SEM models. This result indicates that the SDM model has a better fitting effect. Therefore, the regression results of the bidirectional fixed SDM model are analyzed.

The estimated coefficients of the explanatory variables are all positive, indicating that the construction of urban green spaces positively affects the economic growth of the city. At the same time, the estimated coefficient variables of the control show that the regression coefficient of UGR is significantly positive. If the urban green land construction level increases by 1%, the economic level of surrounding cities increases by 0.3085. The regression coefficient of JS is significantly positive, and the urban green land construction level increases by 0.3085. An increase of 1% will increase the economic level of surrounding cities by 0.016. The regression coefficient of GS is significantly positive, and, if the urban green land construction level increases by 1%, the economic level of surrounding cities will increase by 0.065. The regression coefficient of FDI is significantly positive. For every 1% increase in land construction level, the economic level of surrounding cities will increase by 0.034. The regression coefficient of LB is significantly positive, and, for every 1% increase in urban green land construction level, the economic level of surrounding cities will increase by 0.058. The regression coefficient of HC is significantly positive, and, for every 1% increase in urban green land construction level, the economic level of surrounding cities will increase by 0.016. The regression coefficient of URB is significantly positive, and, for every 1% increase in the level of urban green land construction, the economic level of surrounding cities will increase by 0.048.

The explanatory power of the regression coefficients, the spatial effects, are decomposed. Direct and indirect effects are used to represent the influence of explanatory variables on explained variables. The results are shown in Table 6.

The direct effect of urban green space construction on economic growth is 0.092, which is significant at the 1% level. This means that, for every 1% increase in the level of green space construction in a city, the economy will grow by 0.092, while the indirect effect is 0.144 at a significance level of 5%. This indicates that, for every 1% increase in the level of urban green space construction, the economy of surrounding cities will increase by 0.144. These results show that constructing urban green space optimizes the social industrial structure, gradually eliminates related industries with outdated technologies, and leaves a large scope for future economic development, thus further stimulating the sustainable growth of the urban economy. When the green construction level of a city is high, the city’s industries may choose to transfer to surrounding cities and improve the economic level. Therefore, a city’s green space construction rate will have a positive spatial spillover effect on the economy of neighboring cities.

The direct effect of the labor force on economic growth is 0.073, which is significant at the 5% level, indicating that, for every 1% increase in the labor force, the economy will grow by 0.073. The indirect effect is 0.110, indicating that, for every 1% increase in the labor force, the economy of surrounding cities will increase by 0.110. However, this is not significant, which is because the total amount of labor is limited, and an increase in labor in one city will lead to an increase in the cost of recruiting talents in a different city.

The direct effect of urban green space construction on economic growth is 0.092, which is significant at the 1% level, indicating that, for every 1% increase in the degree of urbanization, the economy will grow by 0.092. The indirect effect is 0.116, which is significant at the 10% level, indicating that, for every 1% increase in the degree of urbanization, the economy of surrounding cities will increase by 0.116.

### 4.4. Robustness Check

To test the robustness of the research results, the values were re-estimated with different explanatory variables. The lag period of urban green space construction was used as the core explanatory variable, and the urban economic growth space overflow was lagged by one period to alleviate the endogeneity problem caused by mutual causation. Because data on the urban green space construction and economic level in 2000 are available, the sample size has not decreased, and Table 7 reports the corresponding estimation results. The direct effect results show that the construction of urban green spaces significantly promotes urban economic growth. The indirect effect results show that the impact of urban green space construction in other cities on local economic growth is also significantly positive. The estimation results also show that not only does current green space construction promote the current economic growth but also that the improvement of the previous green space construction can promote economic growth. This result indicates that, through improvement measures, green space construction can continue to enhance economic growth. This reflects the pull effect of growth. In conclusion, the baseline regression results did not change substantially, indicating that the original model settings and regression estimation results are robust (Table 8).

## 5. Conclusions

This paper uses the spatial panel data of 31 provincial capital cities in China from 2001 to 2020 to construct a spatial econometric model. Then, the model is used to empirically test the impact of urban green space construction rate on economic growth from the perspective of spatial interaction. The following conclusions can be drawn: (1) from the perspective of changes in the spatial distribution of green space completion rates, green space construction hotspot cities have always been concentrated in the east, while cold-spot cities have gradually migrated westward from the central region, with obvious spatial evolution characteristics; (2) from the perspective of time changes, from 2001 to 2010, since the growth rate of green space in the built-up areas of key cities far exceeded that of surrounding cities, there was a large gap in green space construction between cities, so the spatial correlation of urban green space construction was unstable. From 2011 to 2020, Moran’s I index showed a slow upward trend. During this period, the level of green space in urban built-up areas was generally high, at a low level, and in a balanced state; (3) through spatial measurement, it was found that the construction of urban green space can significantly improve the urban environment, attract foreign investment, gather talents, and optimize the industrial structure, thereby effectively improving the economic level of the city. At the same time, there is a spatial spillover effect to drive the economic development of surrounding cities. To sum up, urban green space construction plays an increasingly important role in promoting the sustainable development of the urban economy.

Based on the above research results, the following policy suggestions are put forward: (1) the construction level of urban green space in eastern cities is relatively high, and there is little space to use the improvement in the urban green space rate to stimulate economic development, so it is necessary to shift the construction of urban green spaces from the pursuit of high quantity to the creation of high quality green spaces there. However, there is still significant room for improvement in green space construction in the central and western regions. On the basis of taking into account the quality of urban green space, urban green space should be reasonably increased in the newly added urban construction land, and land consolidation should be carried out in the existing urban construction land to revitalize the urban construction land. (2) The government can appropriately encourage foreign capital to enter the field of urban ecological green space construction, which can accelerate the mutual transformation of various elements in the urban greening industry and form a domestic industrial structure that matches the international environment and improves the rationalization of the greening industry structure; at the same time, the government can encourage enterprises undertaking greening project construction to absorb foreign advanced design concepts and advanced production technology and improve the technical level and service efficiency of urban ecological green space construction; (3) in the future, in terms of urban ecological green space regarding construction planning ideas, with the gradual transformation of the primary and secondary industries of the city to the tertiary industry, the government should withdraw from the planning and construction of urban ecological green space projects and complement the functions of the private sector in order to maintain the quality of project construction management.

## Figures and Tables

**Table 1:** Description of related variables.

	Variable	Symbol	Description
Explained variable	Urban economic growth	lnGDP	Logarithm of gross regional product
Core explanatory variables	Urban green space construction	UGR	Green space rate in built-up area
Control variables	The area of built-up area	JS	Area of municipal utilities and areas with basic public facilities
	Governmental expenditure	GS	Payments made by the government to perform functions and obtain required goods and services
	Foreign direct investment	FDI	Funds borrowed by state-owned enterprises from abroad
	Labor	LB	Total employment in the city
	Human capital level	HC	Average level of human capital possessed by the labor force in a certain area
	Urbanization level	URB	Ratio of urban population to total population

**Table 2:** Spatial evolution trend from 2001 to 2020.

Cities	Urban Economic Growth (*lnGDP*)		Green Space Rate of the Built-Up Area (*UGR*)	
	2001	2020	2001	2020
Chengdu	27	35	8	8.6
Guangzhou	18	40	12.2	8.9
Guiyang	18	40	5.1	6.6
Hohhot	17	38	3.9	5.5
Lhasa	12	21	3.6	5.7
Nanning	22	31	4.2	6.0
Shijiazhuang	14	30	5.7	6.6
Wuhan	26	34	9.7	12.2
Beijing	23	39	10.7	10.8
Changchun	19	23	6.4	6.2
Changsha	15	31	6.2	8.9
Chongqing	20	42	8.5	9.2
Fuzhou	20	43	7.8	7.4
Haikou	17	34	4.2	5.3
Hangzhou	25	32	9.2	8.5
Harbin	18	23	7.2	6.5
Hefei	21	24	4.8	7.6
Jinan	19	39	7.4	7.2
Kunming	19	30	6.8	6.7
Lanzhou	17	32	4.1	5.8
Nanchang	16	29	7.4	7.7
Nanjing	26	43	8.7	9.4
Shanghai	24	33	13.5	10.2
Shenyang	13	20	8.2	7.0
Taiyuan	13	23	5.3	7.2
Tianjin	15	32	4.7	5.8
Urumqi	11	31	3.6	5.5
Xi’an	25	26	6.5	6.8
Xining	12	30	4.6	5.1
Yinchuan	19	42	4.5	5.3
Zhengzhou	13	33	6.2	8.3

**Table 3:** Spatial evolution trend of urban green space construction.

Year	Low Level	Medium Level	High Level
2001	Taiyuan; Xi’an; Changsha; Nanchang; Shijiazhuang; Xining; Urumqi; Zhengzhou; Tianjin; Lhasa; Shenyang	Kunming; Haikou; Changchun; Guangzhou; Chongqing; Jinan; Guiyang; Yinchuan; Fuzhou; Harbin; Lanzhou; Hohhot	Shanghai; Beijing; Nanjing; Hefei; Hangzhou; Nanning; Chengdu; Wuhan
2020	Hefei; Lhasa; Harbin; Changchun; Taiyuan; Xi’an; Shenyang	Lanzhou; Wuhan; Changsha; Nanchang; Shijiazhuang; Xining; Urumqi; Zhengzhou; Chengdu; Kunming; Nanning; Haikou; Tianjin; Shanghai; Hangzhou	Guangzhou; Hohhot; Jinan; Guiyang; Yinchuan; Chongqing; Fuzhou; Beijing; Nanjing

**Table 4:** Spatial evolution trend table of urban economic growth.

Year	Low Growth	Medium Growth	High Growth
2001	Lhasa; Hohhot; Yinchuan; Lanzhou; Xining; Urumqi; Nanning; Haikou; Tianjin; Hefei; Shijiazhuang; Guiyang; Taiyuan	Chongqing; Harbin; Jinan; Fuzhou; Changchun; Zhengzhou; Xi’an; Changsha; Kunming; Nanchang	Shanghai; Beijing; Guangzhou; Chengdu; Hangzhou; Wuhan; Nanjing; Shenyang
2020	Lhasa; Hohhot; Yinchuan; Lanzhou; Xining; Urumqi; Nanning; Haikou; Tianjin	Jinan; Hefei; Fuzhou; Xi’an; Kunming; Changchun; Shenyang; Nanchang; Shijiazhuang; Harbin; Guiyang; Taiyuan	Shanghai; Beijing; Guangzhou; Chongqing; Chengdu; Hangzhou; Wuhan; Nanjing; Zhengzhou; Changsha

**Table 5:** Global Moran’s I index of main variables over the past decades.

Year	Urban Economic Growth (*lnGDP*)	Green Space Rate of the Built-Up Area (*UGR*)
2001	0.275 *** (2.890)	0.192 ** (2.014)
2002	0.279 *** (2.929)	0.242 ** (2.539)
2003	0.283 *** (2.969)	0.174 * (1.826)
2004	0.298 *** (3.127)	0.162(1.700)
2005	0.287 *** (3.008)	0.186 * (1.952)
2006	0.281 *** (2.945)	0.217 ** (2.277)
2007	0.286 *** (3.001)	0.230 ** (2.413)
2008	0.284 *** (2.977)	0.184 * (1.930)
2009	0.269 *** (2.827)	0.195 * (2.046)
2010	0.272 *** (2.850)	0.241 ** (2.528)
2011	0.210 ** (2.203)	0.189 * (1.983)
2012	0.212 ** (2.207)	0.178 * (1.855)
2013	0.212 ** (2.209)	0.216 ** (2.251)
2014	0.216 ** (2.221)	0.223 ** (2.293)
2015	0.218 ** (2.227)	0.232 ** (2.370)
2016	0.219 ** (2.230)	0.236 ** (2.403)
2017	0.219 ** (2.232)	0.245 ** (2.495)
2018	0.220 ** (2.233)	0.249 ** (2.527)
2019	0.223 ** (2.242)	0.258 ** (2.594)
2020	0.214 ** (2.215)	0.249 ** (2.577)

Note: * represents a 10% significance level, ** represents a 5% significance level, and *** represents a 1% significance level.

**Table 6:** Estimated results of spatial econometric models.

Variable	SDM	SAR	SEM
UGR	0.085 *** (2.982)	0.078 *** (2.736)	0.064 ** (2.245)
JS	0.016 ** (2.049)	0.015 * (1.980)	0.012 (1.192)
GS	0.065 *** (4.341)	0.059 *** (3.941)	0.043 *** (2.872)
FDI	0.034 *** (2.643)	0.032 ** (2.488)	0.031 ** (2.410)
LB	0.058 *** (3.481)	0.049 ** (3.245)	0.055 ** (3.426)
HC	0.016 ** (2.128)	0.015 ** (1.995)	0.012 (1.596)
URB	0.048 *** (3.222)	0.046 *** (3.087)	0.043 ** (2.886)
W*UGR	0.012 (0.899)		
W*JS	0.019 (1.423)		
W*GS	0.015 (1.124)		
W*FDI	−0.024 (−1.045)		
W*LB	−0.021 (−0.989)		
W*HC	−0.026 (−1.122)		
W*URB	0.015 (1.120)		
Adj_R^2^	0.688	0.649	0.522
Log-likelihood	760.554	744.131	726.146
Wald_ spatial_ lag	15.320 **	0.021	
LR_ spatial _lag	16.482 **	0.028	
Wald_ spatial_ error	36.002 **	0.000	
LR_ spatial_ error	35.101 **	0.000	

Note: the values in parentheses below coefficients are their standard errors; *, **, and *** represent the significance levels of 1%, 5%, and 10%, respectively.

**Table 7:** Direct, indirect, and spatial effects representing the influence of explanatory variables.

Variable	Direct Effect	Space Spillover Effect	Total Effect
UGR	0.092 *** (2.954)	0.144 ** (2.156)	0.236 ** (2.452)
JS	0.042 (1.349)	0.056 (0.838)	0.098 (1.018)
GS	0.088 *** (2.826)	0.108 (1.617)	0.196 ** (2.036)
FDI	0.085 *** (2.729)	0.116 * (1.837)	0. 201 ** (2.088)
LB	0.073 ** (2.344)	0.110 (1.647)	0.183 ** (1.901)
HC	0.033 (1.060)	0.081 (1.213)	0.114 (1.184)
URB	0.094 *** (3.018)	0.116 * (1.847)	0.210 ** (2.182)

Note: the values in parentheses are the standard errors of coefficients; *, **, and *** represent the significance levels of 1%, 5%, and 10%, respectively.

**Table 8:** Robustness test.

Variable	Direct Effect	Space Spillover Effect	Total Effect
$UGR_{t-1}$	0.101 *** (3.243)	0.245 *** (3.668)	0.346 *** (3.595)
JS	0.051 (1.638)	0.069 (1.033)	0.120 (1.247)
GS	0.092 *** (2.954)	0.116 * (1.838)	0.208 ** (2.161)
FDI	0.094 *** (3.018)	0.143 **(2.141)	0. 237 ** (2.462)
LB	0.077 ** (2.472)	0.115 * (1.822)	0.192 ** (1.995)
HC	0.027 (0.867)	0.076 (1.138)	0.103 (1.070)
URB	0.086 *** (2.761)	0.114 * (1.807)	0.200 ** (2.078)

Note: the values in parentheses are the standard errors of coefficients; *, **, and *** represent the significance levels of 1%, 5%, and 10%, respectively.

## Data Availability

The data that support the findings of this study are available from the corresponding author upon reasonable request.
